# Dysregulation of *In Vitro* Decidualization of Human Endometrial Stromal Cells by Insulin via Transcriptional Inhibition of Forkhead Box Protein O1

**DOI:** 10.1371/journal.pone.0171004

**Published:** 2017-01-30

**Authors:** Dorina Ujvari, Ivika Jakson, Shabnam Babayeva, Daniel Salamon, Bence Rethi, Sebastian Gidlöf, Angelica Lindén Hirschberg

**Affiliations:** 1 Department of Women’s and Children’s Health, Karolinska Institutet, Stockholm, Sweden; 2 Department of Obstetrics and Gynecology, Karolinska University Hospital, Stockholm, Sweden; 3 Department of Obstetrics and Gynecology II, Azerbaijan Medical University, Baku, Azerbaijan; 4 Department of Microbiology, Tumor and Cell Biology, Karolinska Institutet, Stockholm, Sweden; 5 Department of Medicine, Karolinska University Hospital, Stockholm, Sweden; 6 Department of Clinical Science, Intervention and Technology, Karolinska Institutet, Stockholm, Sweden; South China Agricultural University, CHINA

## Abstract

Insulin resistance and compensatory hyperinsulinemia are characteristic features of obesity and polycystic ovary syndrome, and both are associated with reduced fertility and implantation. There is little knowledge about the effect of insulin on the decidualization process and previous findings are contradictory. We investigated the effect of insulin on the regulation of forkhead box protein O1 (*FOXO1*), one of the most important transcription factors during decidualization. Endometrial stromal cells were isolated from six healthy, regularly menstruating women and decidualized in vitro. Gene expression levels of six putative *FOXO1* target genes (including insulin-like growth factor binding protein-1 (*IGFBP1*) and prolactin (*PRL*)) were measured with Real-Time PCR following *FOXO1* inhibition or insulin treatment. PI3K inhibition was used to identify the possible mechanism behind regulation. Subcellular localization of *FOXO1* was analyzed with immunofluorescence. All the genes (*IGFBP1*, *CTGF*, *INSR*, *DCN*, *LEFTY2*), except prolactin, were evaluated as *FOXO1* target genes in decidualizing stromal cells. Insulin caused a significant dose-dependent inhibition of the verified *FOXO1* target genes. It was also demonstrated that insulin regulated *FOXO1* target genes by transcriptional inactivation and nuclear export of *FOXO1* via *PI3K* pathway. However, insulin did not inhibit the morphological transformation of endometrial stromal cells via transcriptional inactivation of *FOXO1*. This study provides new insights on the action of insulin on the endometrium via regulation of *FOXO1*. It is suggested that hyperinsulinemia results in dysregulation of a high number of *FOXO1* controlled genes that may contribute to endometrial dysfunction and reproductive failure. Our findings may illuminate possible reasons for unexplained infertility.

## Introduction

Insulin resistance and compensatory hyperinsulinemia are common features of obesity and polycystic ovary syndrome (PCOS), which are both associated with reduced fertility and pregnancy complications, including impaired decidualization and implantation, miscarriage, gestational diabetes, preeclampsia, intra-uterine growth restriction and preterm birth [1–6].

It is well-known that insulin resistance and hyperinsulinemia could contribute to hyperandrogenism and ovarian dysfunction [7]. The endocrine and metabolic abnormalities associated with PCOS and obesity may also affect endometrial function and receptivity [8]. However, very little is known about the effects of insulin on endometrial function. We have previously shown that lifestyle intervention in obese women with PCOS results in lower fasting insulin levels, enhanced insulin signaling in the endometrium and improved menstrual cyclicity [9].

Decidualization is differentiation of endometrial stromal fibroblasts into secretory epithelioid decidual cells that is essential for implantation and normal pregnancy. The decidual process is characterized by the expression of a variety of phenotypic markers. The most well-known genes are prolactin (*PRL*) and insulin-like growth factor binding protein-1 (*IGFBP1*) [10].

There is little knowledge about the effect of insulin on the decidualization process and previous findings are contradictory. It has been demonstrated that insulin down-regulates *IGFBP1* in decidualizing human endometrial stromal cells [11, 12], whereas *PRL* production is stimulated [13]. Thus, the effect of insulin on decidualization needs to be further explored.

The evolutionary conserved transcription factor forkhead box protein O1 (*FOXO1*) is one of the earliest induced transcription factors in the decidualization process of human endometrial stromal cells [10, 14, 15]. It was demonstrated that among half a thousand of genes, *PRL* and *IGFBP1* were aberrantly expressed upon *FOXO1* knockdown in decidualizing human endometrial stromal cells supporting the importance of *FOXO1* during differentiation of endometrial stromal cells [16]. Furthermore, overexpression of *FOXO1* was found to induce a decidual morphology in endometrial stromal cells, suggesting a cardinal role of *FOXO1* in acquisition of the epitheloid phenotype during decidualization [17]. Previous studies have suggested that *FOXO1* activity can be regulated by insulin through the *PI3K* pathway [18].

In this in vitro study, we aimed to investigate *FOXO1* transcriptional activity as a possible mechanism by which insulin modulates decidualization of human endometrial stromal cells.

## Materials and Methods

### Subjects

Six regularly cycling, non-smoking healthy volunteers underwent collection of endometrial biopsy under local anesthesia using a suction curette (Pipet Curet, CooperSurgical, USA) at cycle day 5–9. All participants were between 18–35 years and had a body mass index ranging 19–28. Hormonal medication was not allowed at least three months prior to biopsy sampling. No women had any current chronic disease, endocrine disorder, or continuous medication. One woman had had an uneventful pregnancy with normal birth at term and another woman had had one miscarriage prior to the study, she later gave birth at term after IVF treatment. No cause of the infertility could be determined. The other four women had not been pregnant. Serum levels of follicle-stimulating hormone (FSH), luteinizing hormone (LH), estradiol-17β (E2), total testosterone sex hormone-binding globulin (SHBG) and fasting glucose were determined as described previously [9, 19]. FSH (5.7±0.8 IU/l), LH (5.9±1.4 IU/l), E2 (253 (115–466 pmol/l)), total testosterone (0.9±0.3 nmol/l), SHBG (69.2±26.9 nmol/l) and fasting glucose levels (4.8±0.5 mmol/l) were in the normal range for all of them (values presented are means±SD or medians and interquartile ranges, depending on the distribution).

### Ethical approval

All women gave their written informed consent and the local ethics committee approved the study at the Regional Ethical Committee in Stockholm (Dnr 2008/865-32).

### Isolation of human endometrial stromal cells

Isolation of endometrial stromal cells was carried out after modification of a method previously described [20, 21]. Briefly, the endometrial tissues were transferred in Ham’s F-10 Nut Mix medium (Thermo Fischer Scientific, USA) supplemented with 0.2% penicillin-streptomycin (Sigma-Aldrich, USA) and 20% of HI-FBS (Sigma-Aldrich, USA) on ice. After washing, the tissues were minced with a scalpel to approximately 1 mm^3^ pieces, and filtered through a 100 μm pore size cell strainer (Falcon BD Biosciences, Belgium) to remove red blood cells. After sedimentation (140 g, 5 min, 4°C), tissues were digested with collagenase III (Worthington, USA) (1 mg/ml) supplemented with DNase I (40 μg/ml) (Sigma-Aldrich, USA) in PBS at 37°C for 2–2.5 h in a shaking thermostat to obtain single stromal cells. After sedimentation (120g, 10 min, 4°C), the cell suspensions were washed with Ham`s F-10 Nut mix. The cell suspensions were filtered through a 40μm cell strainer (Falcon BD Biosciences, Belgium), allowing single stromal cells to pass through, while glandular tubules were restrained. Stromal cells were sedimented (120g, 10 min, 4°C) and then frozen in freezing mixture (Gibco) and kept on -150°C until use. Freezing did not affect cell viability significantly (data not shown). Cell viability was monitored using Trypan blue staining. Purity of stromal cells was ensured by sequential culturing and assessed by cytokeratin and CD10 staining for epithelial and stromal cells, respectively.

### Culture conditions

Endometrial stromal cells, 10^5^/well, were seeded in 6-well Costar plates (Sigma-Aldrich, USA) and cultured in DMEM/F12-Glutamax (Thermo Fischer Scientific, USA) supplemented with 10% fetal bovine serum and 0.2% penicillin-streptomycin until ~80% confluency. *In vitro* decidualization was performed using a well-established procedure as previously described [22–24]. Briefly, media was changed to DMEM/F12 without phenol red (Thermo Fischer Scientific, USA), supplemented with 2% charcoal stripped fetal bovine serum (Sigma-Aldrich, USA) and 0.2% penicillin-streptomycin. In order to investigate the kinetics of decidualization, we treated the cells with 1 μM medroxyprogesterone-17-acetate (MPA) (Sigma-Aldrich, USA) and 1 mM N^6^, 2`-O-dibutyryladenosine cAMP (db-cAMP) (Sigma-Aldrich, USA) for 3 and 6 days. To clarify the contribution of each of MPA and db-cAMP to induce decidualization, endometrial stromal cells were treated with 1 μM MPA, 1 mM db-cAMP or combined treatment of MPA and db-cAMP for 6 days. Furthermore, to investigate the effect of insulin on decidualization and *FOXO1* target genes, cells were treated with 1 μM MPA and 0.5 mM db-cAMP in the presence or absence of 5, 50 or 500 nM insulin (Sigma-Aldrich, USA).

To test if wortmannin, a *PI3K* inhibitor, can block the effect of insulin, stromal cells were pre-decidualized for 3 days and then pre-treated according to previous publications [12, 25] with 500 nM wortmannin (Sigma-Aldrich, USA) for 1 hour prior to addition of 100 nM insulin for 2 days along with decidualization agents db-cAMP and MPA. To evaluate the involvement of *FOXO1* in the regulation of a particular gene, stromal cells were pre-decidualized for 3 days and then treated with 100 nM AS1842856 (Merck Millipore, Germany), a *FOXO1* inhibitor, for 2 more days in the presence of decidualization agents db-cAMP and MPA. The culture media was renewed every 3 days. Depending on the experiment, cells were harvested for RNA isolation after 3, 5 or 6 days.

### RNA isolation, cDNA synthesis and real-time PCR

Total RNA was extracted and 1 μg RNA was subjected to cDNA synthesis as described previously [9]. The optimal amount of cDNA for each gene was tested with a dilution series subjected to Real-Time PCR. cDNAs of all the investigated genes were then measured with Real-Time PCR (TaqMan or SybrGreen methods) along with the housekeeping gene ribosomal protein L13a (RPL13A), using the same amount of cDNA (10–40 ng) for both target and housekeeping gene, depending on the abundance of the target gene. The employed TaqMan assays (Thermo Fischer Scientific, USA) and oligonucleotides (Sigma-Aldrich, USA) are listed in S1 and S2 Tables. The reactions were run in a StepOnePlus^TM^ Real-Time PCR System (Thermo Fischer Scientific, USA). Gene expression levels of putative *FOXO1* target genes (*PRL*, *IGFBP1*, connective tissue growth factor (*CTGF*), insulin receptor (*INSR*), decorin (*DCN*) and left-right determination factor 2 (*LEFTY2*)) and a forkhead box protein M1 (*FOXM1*) target gene, signal transducer and activator of transcription 3 (*STAT3*), known to be up-regulated upon decidualization were determined using the ΔΔC_T_ method. The PCR efficiency with all amplicons was 90–100% and all determinations were performed in triplicate.

### Immunofluorescence

In order to evaluate the effect of insulin on the subcellular localization of *FOXO1* and if this effect is conveyed by PI3K, endometrial stromal cells were plated into culture chambers at a density of 2x10^4^ and cultured until ~70% confluency. The cells were pre-decidualized for 3 days and then pre-treated with 500 nM wortmannin (Sigma-Aldrich, USA) for 1 hour prior to treatment with 100 nM insulin. Since it has been published earlier that *PI3K* signaling led to phosphorylation, nuclear exclusion and proteasomal degradation of *FOXO1* in some other cell types [26–31], we chose a shorter, 3h insulin treatment, in order to be able to capture the change in subcellular localization in response to insulin, prior to an eventual proteasomal degradation of *FOXO1*. After the treatment cells were fixed with 3.7% paraformaldehyde in PBS for 20 min and permeabilized by treatment with 0.1% Triton X-100 in PBS for 30 min. After blocking with 3% BSA, 0.1% Tween 20 in PBS for 1 h, cells were incubated with anti-*FOXO1* antibody (Cell Signaling Technology, USA) using 1:200 dilution, followed by staining with Alexa Fluor 488 anti-mouse secondary antibody. Pictures were acquired using 630x magnification with a Zeiss AxioVert 40 CFL microscope equipped with a Zeiss AxioCAM MRM digital camera and the AxioVision software.

### Statistical analysis

All values are presented as medians and ranges (min-max). Statistical analysis was performed including all the six subjects by the Friedman test followed by Dunn`s multiple comparison, where each treatment group was tested against the untreated group. In the *PI3K* inhibitory experiments, the insulin treated groups were compared with their respective controls (decidual, decidual + solvent or decidual + PI3K inhibitor). Normality of the data was tested with the Kolgomorov-Smirnov test. P < 0.05 was considered as significant.

## Results

### db-cAMP and MPA induce decidualization in human primary endometrial stromal cells

To investigate the effect of db-cAMP and MPA on human primary endometrial stromal cells, we treated the cells with MPA, db-cAMP or their combination for 6 days. We found that db–cAMP alone induced morphological transformation of the cells and up-regulated decidualization markers, whereas MPA did not. The combined use of MPA and db-cAMP also induced morphological change of the cells and enhanced up-regulation of decidualization markers compared to db-cAMP alone (S1 Fig). Furthermore, we investigated the kinetics of decidualization in response to MPA and db-cAMP and found that decidualization was apparent after 3 days, but was even more enhanced after 6 days (S2 Fig).

### Several putative *FOXO1* target genes are down-regulated upon the inhibition of *FOXO1* in decidualizing human endometrial stromal cells

To establish whether *FOXO1* regulates *PRL*, *IGFBP1*, *CTGF*, *INSR*, *DCN*, and *LEFTY2* gene expression levels in decidualizing endometrial stromal cells, we treated cells with a *FOXO1* inhibitor (AS1842856) in the presence of the decidualizing agents MPA and db-cAMP. *FOXO1* inhibition in decidualizing endometrial stromal cells resulted in down-regulation of *IGFBP1* (Fold change (FC) = 0.13), *CTGF* (FC = 0.2), *INSR* (FC = 0.2), *DCN* (FC = 0.43) and *LEFTY2* (FC = 0.005), whereas *PRL* (FC = 1.1) was not significantly changed (Table 1). These results indicate that *FOXO1* regulates the above genes, with the exception of *PRL*, in human decidualizing stromal cells.

**Table 1:** Comparison of relative levels in the presence or absence of FOXO1 inhibitor in decidualizing endometrial stromal cells.

Gene	Control	+ solvent	+ FOXO1 inhibitor (100 nM) nM)
***PRL***	86.3 (33.3–113.8)	95.8 (35.2–142.5)	108.8 (51.8–172.0)^NS^
***IGFBP1***	27.9 (6.8–100.0)	32.7 (5.9–118.4)	5.4 (1.2–9.7)^*^
***CTGF***	72.1 (20.3–172.5)	70.8 (17.5–134.0)	12.4 (8.0–16.1)^**^
***INSR***	49.7 (30.0–100.0)	55.3 (30.9–110.0)	12.7 (4.7–29.9)^***^
***DCN***	77.3 (26.3–492.6)	75.5 (37.6–399.0)	17.5 (11.3–250.1)^**^
***LEFTY2***	52.9 (18.6–100.0)	59.8 (19.7–152.3)	0.3 (0.1–0.4)^****^

Values presented are medians and ranges (min-max).

* = p<0.05

** = p<0.01

*** = p<0.001

**** = p<0.0001 and NS = not significant represent the significance levels between control and *FOXO1* inhibitory treatments. Differences between solvent and control treatment were not significant.

### Insulin inhibits *FOXO1* induced target genes in decidualizing human endometrial stromal cells

**Fig 1:**
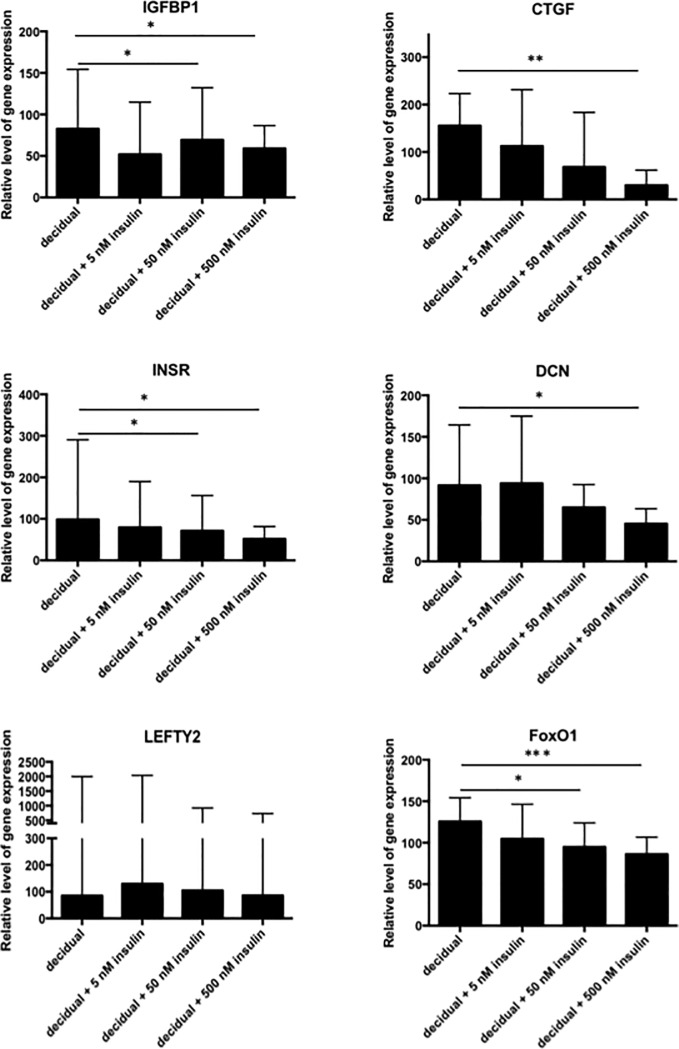
Relative gene expression levels in response to different concentrations of insulin in decidualizing endometrial stromal cells. Relative gene expression levels of *IGFBP1*, *CTGF*, *INSR*, *DCN*, *LEFTY2*, and *FOXO1* in response to 5, 50 and 500 nM insulin in *in vitro* decidualized human endometrial stromal cells after 6 days. The values presented are medians and ranges (min-max). * = p < 0.05; ** = p < 0.01; *** = p < 0.001 in comparison to the control value.

To test if insulin regulates the verified *FOXO1* target genes, proliferative phase derived stromal cells were decidualized in the presence or absence of increasing concentrations of insulin, and relative gene expression levels of *FOXO1* and some of its target genes were measured. *FOXO1*, *IGFBP1*, *INSR*, *DCN* and *LEFTY2* were significantly up-regulated upon decidualization, while *CTGF* was not significantly changed (S3 Fig). On the other hand, *IGFBP1*, *CTGF*, *INSR* and *DCN* were significantly down-regulated by insulin in a dose dependent fashion, while *LEFTY2* was not significantly changed (Fig 1). These results indicate that increasing concentrations of insulin in decidualizing endometrial stromal cells alter the action of *FOXO1* on its target genes. In contrast, to the inhibitory effect of insulin on the verified *FOXO1* target genes, *PRL* expression was significantly stimulated by the highest dose of insulin (FC = 3.0; P<0.05). To determine if *FOXO1* itself is regulated by insulin on a transcriptional level we measured *FOXO1* gene expression level in response to insulin. We found that insulin down-regulated *FOXO1* gene expression level in a dose dependent fashion meaning that insulin regulates *FOXO1* at a transcriptional level in decidualizing endometrial stromal cells (Fig 1). To determine if insulin has similar effect on other members of forkhead box proteins of importance for decidualization of endometrial stromal cells, we measured the gene expression level of *STAT3*, a well-known target of *FOXM1*. We found that *STAT3* was significantly up-regulated upon decidualization, but was not changed by any of the concentrations of insulin, suggesting that the action of insulin is specific to *FOXO1* in these cells (S4 Fig).

### Inhibition of *FOXO1* is insufficient to prevent morphological transformation of endometrial stromal cells during decidualization

**Fig 2:**
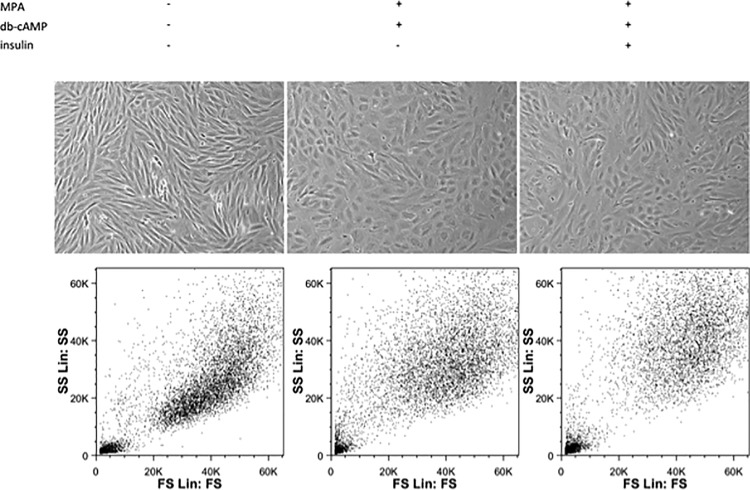
Micrographs and forward and side scatter analysis of undifferentiated stromal and decidualized cells in the absence or presence of insulin. Representative micrographs of undifferentiated stromal and decidualized cells (MPA + db-cAMP) in the absence or presence of insulin were taken using an inverted microscope with 40x magnification. Forward vs side scatter (FCS/SSC) dot plot generated by flow cytometer showing the cell distribution of undifferentiated stromal and decidualized cells in the absence or presence of insulin.

We also investigated the decidualization of endometrial stromal cells with microscopy and flow cytometry in the absence or presence of insulin. We found that despite the significant downregulation of several *FOXO1* target genes, insulin did not inhibit the morphological transformation of spindle-shaped endometrial stromal cells into polygonal epitheloid-like cells (cell size: stromal vs decidual P < 0.05; stromal vs decidual+insulin P < 0.05; decidual vs decidual+insulin not significant (NS); cell granularity: stromal vs decidual P < 0.05; stromal vs decidual+insulin P = 0.0551 (NS); decidual vs decidual+insulin P = 0.0782 (NS)) (Fig 2).

### Insulin regulates *FOXO1* transcriptional activity through *PI3K* in decidualizing human endometrial stromal cells

**Fig 3:**
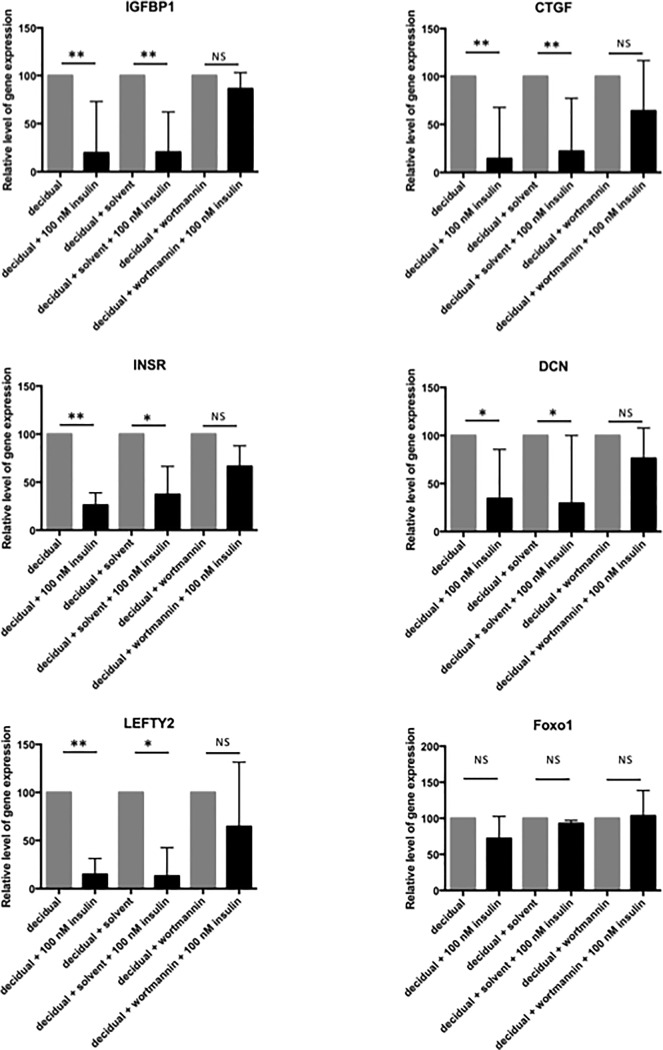
Relative gene expression levels in response to insulin and/or *PI3K* inhibitor in decidualizing endometrial stromal cells. Relative levels of *IGFBP1*, *CTGF*, *INSR*, *DCN*, *LEFTY2* and *FOXO1* in response 500 nM wortmannin, a *PI3K* inhibitor in combination with 100 nM insulin in *in vitro* decidualized human endometrial stromal cells after 4 days. The values presented are medians and ranges (min-max). * = p < 0.05; ** = p < 0.01; in comparison to the appropriate control value.

To investigate if insulin regulates *FOXO1* through the *PI3K* pathway we blocked *PI3K* signaling with wortmannin during the decidualization process in the presence or absence of insulin. It was found that *PI3K* inhibition partially reversed the insulin-induced down-regulation of *IGFBP1*, *CTGF*, *INSR*, *DCN* and *LEFTY2* (Fig 3). However, gene expression levels of *PRL* and *FOXO1* were not significantly influenced by *PI3K* inhibition (Fig 3). These results suggest that regulation of *FOXO1* transcriptional activity by insulin is conveyed by *PI3K* signaling. It must be noted, that *PI3K* inhibition in the absence of insulin did not have any significant effect on the investigated genes (not shown), meaning that PI3K inhibition did not affect the expression of these genes at this stage in decidualizing endometrial stromal cells.

### Insulin induces nuclear export of *FOXO1* in decidualizing human endometrial stromal cells

**Fig 4:**
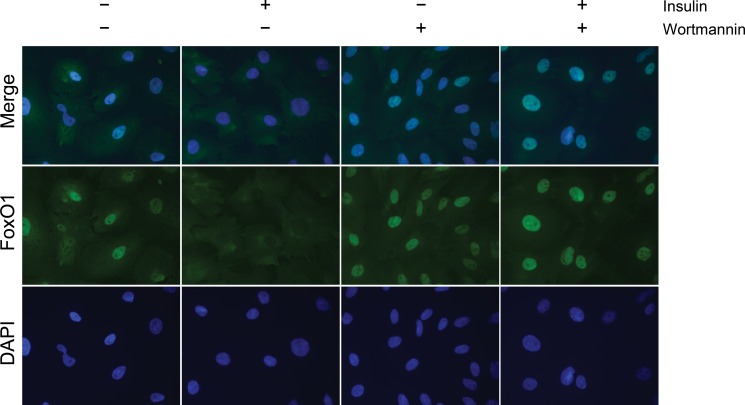
*FOXO1* immunostaining in response to insulin and/or *PI3K* inhibitor. Representative fluorescent micrographs for *FOXO1* (green) and DAPI-stained nucleus (blue) in decidualized cells with 630x magnification. *FOXO1* is located predominantly in the nucleus in the absence of insulin, while exported from the nucleus in the presence of insulin. Wortmannin, a *PI3K* inhibitor has no effect on the subcellular localization of *FOXO1* alone, while in combination with insulin it blocks insulin induced nuclear export of *FOXO1*.

We investigated the subcellular localization of *FOXO1* in decidualizing endometrial stromal cells in response to insulin treatment by fluorescent microscopy. In the absence of insulin, *FOXO1* was predominantly localized within the nucleus (Fig 4). In contrast, insulin treatment rapidly induced the nuclear export of *FOXO1* (Fig 4). To assess if insulin regulation of nuclear export of *FOXO1* is signaled through *PI3K* we blocked this pathway in decidualizing endometrial stromal cells in the presence or absence of insulin. We found that *PI3K* inhibition blocked insulin’s effect on nuclear *FOXO1* export, suggesting that insulin induces nuclear export of *FOXO1* through *PI3K* signaling (Fig 4).

## Discussion

This study is the first to show that insulin alters gene expression of certain decidual markers [14] and other genes, which are up-regulated upon decidualization by regulation of *FOXO1* transcriptional activity. It was demonstrated by the determination of mRNA expression that insulin regulates *FOXO1* target genes through transcriptional inactivation and nuclear export of *FOXO1* in decidualizing primary human endometrial stromal cells. Furthermore, insulin regulates *FOXO1* activity not only on a post-translational level, but also to a minor extent on a transcriptional level. However, insulin does not inhibit the morphological transformation of endometrial stromal cells to decidualized cells.

We demonstrated that MPA and db-cAMP induced decidualization of endometrial stromal cells in agreement with previous observations [22–24]. Furthermore, we evaluated the ability and contribution of MPA and db-cAMP to induce decidualization and found that MPA alone did not induce decidualization markers, but enhanced the effect of db-cAMP remarkably as shown previously [10, 32, 33].

We confirmed *IGFBP1* and *LEFTY2* as *FOXO1* target genes [16], and further demonstrated the regulation of *CTGF*, *INSR* and *DCN* by *FOXO1*, but not *PRL*, in decidualizing endometrial stromal cells. Previous findings on *PRL* regulation by *FOXO1* in decidualizing human endometrial stromal cells have been inconclusive. Buzzio et al showed an up-regulation of *PRL* by *FOXO1* silencing upon decidualization [17]. In contrast, Takano et al reported that silencing of *FOXO1* with siRNA resulted in significant down-regulation of *PRL* in decidualizing endometrial stromal cells [16]. Thus, there is no clear evidence for a consistent regulation of *PRL* by *FOXO1* in these cells.

Here we further show that insulin dose-dependently down-regulates the *FOXO1* target genes *IGFBP1*, *CTGF*, *INSR* and *DCN*, which are up-regulated upon decidualization. In contrast, gene expression of *PRL* was up-regulated by insulin. Our results showing different regulation of *IGFBP1* and *PRL* by insulin are in agreement with others findings. It was previously demonstrated that *IGFBP1* was clearly down-regulated in decidualizing endometrial stromal cells in response to insulin [11, 12], while *PRL* was slightly enhanced [13]. However, investigating the decidual progression in response to insulin using solely *PRL* and *IGFBP1* as markers fails to draw attention to the possible regulation of these genes via transcriptional inactivation of *FOXO1* [16]. This study shows that insulin may influence a high number of genes that are up-regulated upon decidualization via transcriptional inactivation of *FOXO1*, whereas *PRL* seems to be regulated by insulin through a different mechanism.

We found no significant difference in morphological transformation of spindle-shaped stromal cells to polygonal epithelial-like decidual cells by decidualization agents db-cAMP and MPA in the presence or absence of insulin, meaning that insulin does not inhibit decidualization morphologically. Hypothetically this could be explained by an action of *PRL*. Previous publications suggested a critical role of *PRL* in the differentiation of a variety of cell types including endometrial glandular epithelial cells [34–41]. Furthermore, *PRL* was shown to act as an inducer of decidualization of endometrial stromal cells [42, 43]. We could confirm others’ observation on insulin induced up-regulation of *PRL* expression in decidual cells [13]. It is therefore suggested that increased expression of *PRL*, as a differentiation factor could be responsible for the morphological transformation of stromal cells in the presence of insulin. However, the exact role of prolactin in the induction of decidualization markers and morphological transformation of endometrial stromal cells has to be investigated further.

Inhibition of the *PI3K* pathway resulted in a partial reversion of the effect of insulin on the *FOXO1* target genes, suggesting that insulin regulates *FOXO1* transcriptional activity mainly by *PI3K* signaling. The most likely explanation for the incomplete inhibition is that insulin also can induce *PI3K* independent signaling cascades to regulate the investigated *FOXO1* target genes. Alternatively, insulin signaling may also modulate the activity of the protein binding partners of *FOXO1*, which specifically can either activate or repress *FOXO1* target genes [15, 18, 44–52].

We took into consideration the regulation of other forkhead box proteins by insulin in decidualizing endometrial stromal cells. Forkhead box protein O3A (*FOXO3A*) was shown to be down-regulated upon decidualization, whereas forkhead box protein O4 (*FOXO4*) is not expressed in the endometrium [53]. On the other hand, we confirmed the up-regulation of *STAT3* upon decidualization [54], but failed to show its regulation by insulin, suggesting that the effect of insulin was specific to *FOXO1*. It was also shown that inhibition of *FOXM1* could prevent decidualization [54], while insulin could not in our experiments. These observations further suggest that insulin does not inhibit the activity of *FOXM1* in decidualizing stromal cells.

**Fig 5:**
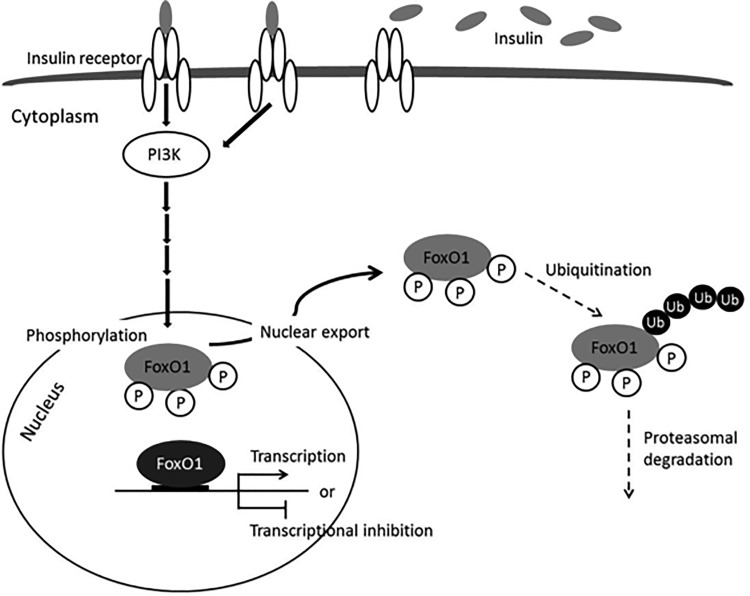
A model on *FOXO1* regulation via insulin-induced phosphorylation-dependent degradation. A model on *FOXO1* regulation through insulin-induced phosphorylation-dependent degradation as suggested in some other cell types: *PI3K* is activated by insulin, the *FOXO* proteins are phosphorylated, resulting in their transcriptional inactivation through their nuclear export and cytoplasmic retention followed by a multistep negative regulation including ubiquitination and proteasomal degradation [26–31].

External stimuli control *FOXO* activity primarily by regulating its subcellular localization [55]. In normal luteal phase endometrium, cAMP induces nuclear accumulation of *FOXO1* protein, while continuous progesterone signaling induces nuclear export of *FOXO1*. The latter is resulting in transcriptional inactivation of a fraction of the total *FOXO1* pool. Fall of progesterone levels in the late luteal phase of the menstrual cycle rapidly leads to re-accumulation of nuclear *FOXO1*, expression of pro-apoptotic *FOXO1* target genes, cell death and menstrual bleeding [33, 56]. It has been demonstrated in a number of other cell types, that when *Akt*, downstream of *PI3K*, is activated the *FOXO* proteins are directly phosphorylated [26–31]. This results in their transcriptional inactivation through their nuclear export, followed by a multistep negative regulation including ubiquitination and proteasomal degradation [29]. Previous studies have also suggested that *FOXO1* activity can be regulated by insulin through the *PI3K* pathway (19) (Fig 5).

Our study is the first to demonstrate that insulin induces transcriptional inactivation of *FOXO1* by its nuclear export via *PI3K* pathway in decidualizing endometrial stromal cells, as shown in other cell types [18]. Although post-translational inhibition of *FOXO* proteins has been well defined in a large diversity of cells, other levels of regulation, such as mRNA expression, has remained largely unexplored. In this study, we were able to show that insulin also down-regulates *FOXO1* mRNA expression in decidualizing endometrial stromal cells. Very limited data is available on transcriptional down-regulation of *FOXO1* by insulin, however it was previously demonstrated that both transcriptional and post-translational regulation share the *PI3K* pathway [57, 58].

Finely tuned decidualization is critical to normal embryo implantation, placentation and maintenance of pregnancy. Disturbances in this process and dysregulation of genes induced upon decidualization may lead to both reduced fertility and adverse pregnancy outcomes, as it was already shown for most of those factors we investigated here [59–63]. Our study suggests an adverse effect of insulin on *FOXO1* regulation during decidualization. Furthermore, despite ovulatory cycles or ovulation induction, hyperinsulinemia in women with PCOS and obesity may still affect the endometrium adversely. Insulin may negatively affect the nuclear re-accumulation of *FOXO1* prior to menstruation that might lead to dysregulation of *FOXO1* induced pro-apoptotic genes. This may contribute to endometrial dysfunction and menstrual disturbances in women with hyperinsulinemic conditions such as PCOS and obesity [64–66]. We have previously shown that reduced insulin levels in obese women with PCOS after lifestyle intervention resulted in improved endometrial insulin signaling and menstrual cyclicity [9]. This indirectly supports the role of *FOXO1* regulation in endometrial breakdown. Our present findings highlight the importance of an increased knowledge of the action of insulin on endometrial function, and may illuminate possible reasons to unexplained infertility. It has to be noted that our aim was to study basic molecular mechanisms associated with hyperinsulinemia only, and not in the context of specific disorders. However, we do expect the general mechanisms to be applicable to many hyperinsulinemic disorders.

In summary, this study is the first to show that insulin can down-regulate the expression of *FOXO1* induced genes in decidualizing primary human endometrial stromal cells. Insulin regulates *FOXO1* activity predominantly on a post-translational level via its nuclear export and to a minor extent on a transcriptional level. We have shown that insulin action on *FOXO1* activity in decidualizing endometrial stromal cells is mainly mediated through the *PI3K* pathway. This study suggests that insulin dysregulates *FOXO1* induced genes upon decidualization, although morphological transformation is not inhibited.

## Supporting Information

S1 Fig Relative gene expression levels in response to MPA, db-cAMP and their combined treatment. A. Representative micrographs of undifferentiated stromal cell and cells in response to MPA (1 μM), db-cAMP (0.5 mM) and their combined treatment after 6 days were taken using an inverted microscope with 40x magnification. B. Relative gene expression levels of *IGFBP1*, *CTGF*, *INSR*, *DCN*, *LEFTY2* and *FOXO1* in response to MPA (1 μM), db-cAMP (0.5 mM) and their combined treatment in endometrial stromal/decidualizing cells after 6 days. The values presented are medians and ranges (min-max). * = p < 0.05 and ** = p < 0.01 in comparison to the control (stromal) value.
(TIF)

S2 Fig Relative gene expression levels in response to combined treatment of MPA and db-cAMP after 3 and 6 days. A. Representative micrographs of undifferentiated stromal and decidualized cells (MPA + db-cAMP) after 3 and 6 days were taken using an inverted microscope with 40x magnification. B. Relative gene expression levels of *IGFBP1*, *CTGF*, *INSR*, *DCN*, *LEFTY2* and *FOXO1* in response to decidualization agents MPA (1 μM) and db-cAMP (0.5 mM) in endometrial stromal/decidual cells after 0, 3 and 6 days. The values presented are medians and ranges (min-max). * = p < 0.05, ** = p < 0.01 and *** = p < 0.001 in comparison to the control (stromal) value.
(TIF)

S3 Fig Relative gene expression levels in response to combined treatment of MPA and db-cAMP after 6 days. Relative gene expression levels of *IGFBP1*, *CTGF*, *INSR*, *DCN*, *LEFTY2* and *FOXO1* in the absence or presence of decidualization agents MPA (1 μM) and db-cAMP (0.5 mM) in endometrial stromal/decidual cells after 6 days. The values presented are medians and ranges (min-max). * = p < 0.05 and *** = p < 0.001 in comparison to the control value.
(TIF)

S4 Fig Relative gene expression level of *STAT3* in response to combined treatment of MPA and db-cAMP and insulin. A. Relative gene expression level of *STAT3* in the absence or presence of decidualization agents MPA (1 μM) and db-cAMP (0.5 mM) in endometrial stromal/decidual cells after 6 days. The values presented are medians and ranges (min-max). * = p < 0.05 in comparison to the control value. B. Relative gene expression level of *STAT3* in response to decidualization agents MPA (1 μM) and db-cAMP (0.5 mM) in the presence or absence of 5, 50 and 500 nM insulin in endometrial stromal cells after 6 days. The values presented are medians and ranges (min-max).
(TIF)

S1 Table TaqMan assay applied in Real-Time PCR. TaqMan assays applied for amplification of prolactin (*PRL*), insulin-like growth factor binding protein-1 (*IGFBP1*) and insulin receptor (*INSR*). *RPL13A* was used as an endogenous control.
(DOCX)

S2 Table Oligonucleotides applied in Real-Time PCR. Forward and reverse oligos applied for amplification of connective tissue growth factor (*CTGF*), decorin (*DCN*) and left-right determination factor 2 (*LEFTY2*). *RPL13A* was used as an endogenous control.
(DOCX)
