# Supplementary figures and images for: Dysregulation of In Vitro Decidualization of Human Endometrial Stromal Cells by Insulin via Transcriptional Inhibition of Forkhead Box Protein O1
Source: PLoS One. 2017 Jan 30;12(1):e0171004. doi: 10.1371/journal.pone.0171004 (PMC5279782; doi:10.1371/journal.pone.0171004)

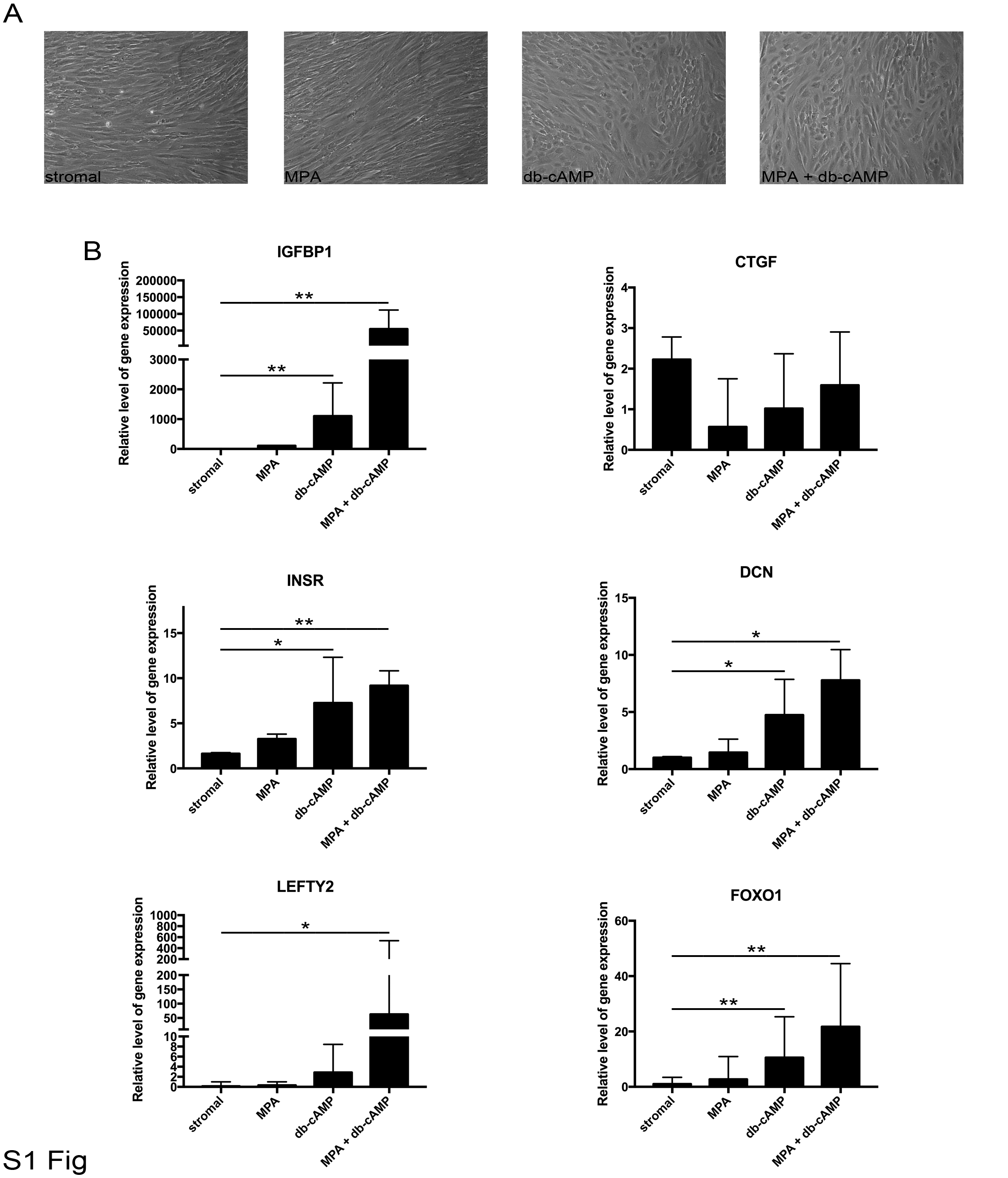

Supplement: S1 Fig — A. Representative micrographs of undifferentiated stromal cell and cells in response to MPA (1 μM), db-cAMP (0.5 mM) and their combined treatment after 6 days were taken using an inverted microscope with 40x magnification. B. Relative gene expression levels of IGFBP1, CTGF, INSR, DCN, LEFTY2 and FOXO1 in response to MPA (1 μM), db-cAMP (0.5 mM) and their combined treatment in endometrial stromal/decidualizing cells after 6 days. The values presented are medians and ranges (min-max). * = p < 0.05 and ** = p < 0.01 in comparison to the control (stromal) value. (TIF) [file pone.0171004.s001.tif]

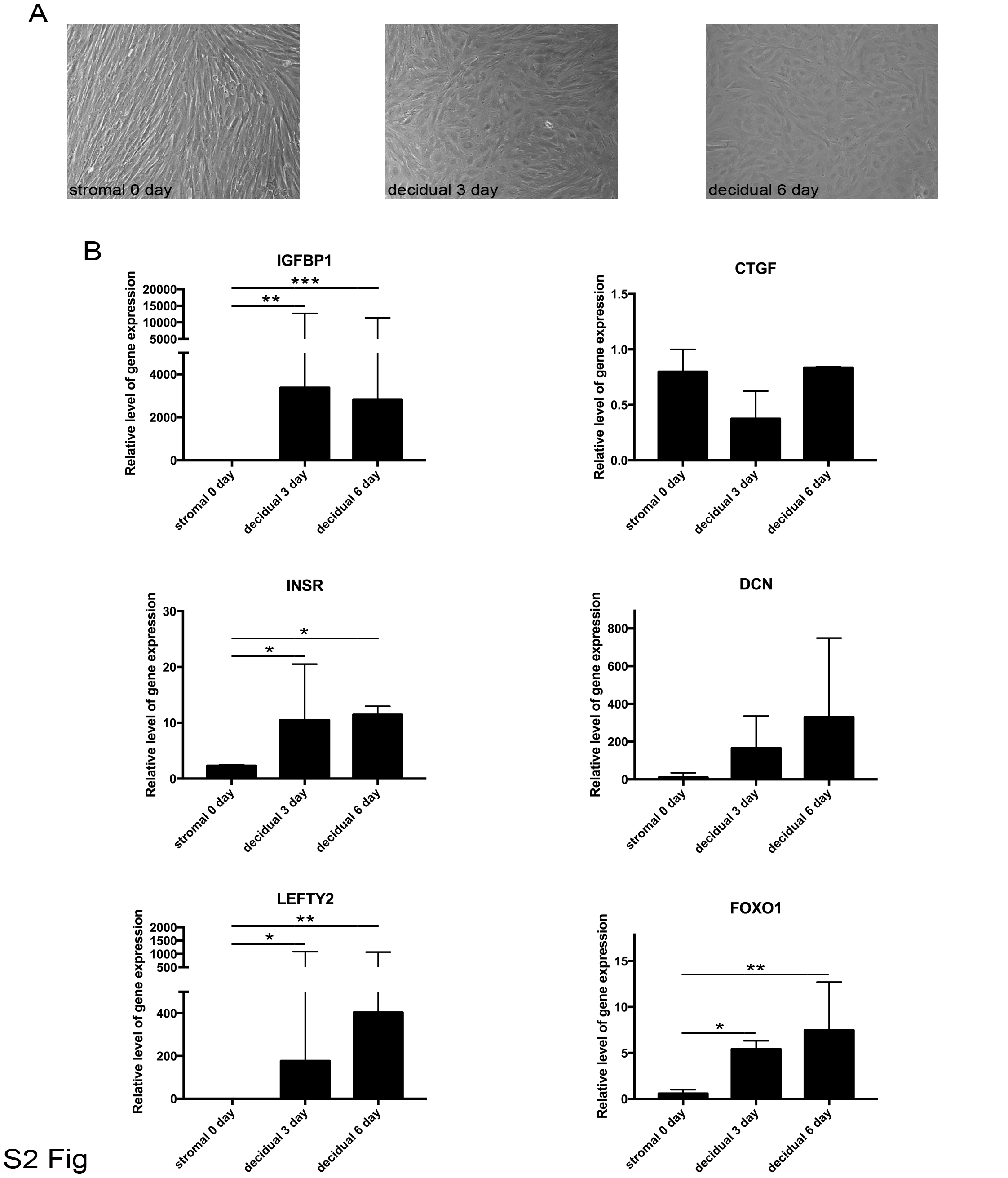

Supplement: S2 Fig — A. Representative micrographs of undifferentiated stromal and decidualized cells (MPA + db-cAMP) after 3 and 6 days were taken using an inverted microscope with 40x magnification. B. Relative gene expression levels of IGFBP1, CTGF, INSR, DCN, LEFTY2 and FOXO1 in response to decidualization agents MPA (1 μM) and db-cAMP (0.5 mM) in endometrial stromal/decidual cells after 0, 3 and 6 days. The values presented are medians and ranges (min-max). * = p < 0.05, ** = p < 0.01 and *** = p < 0.001 in comparison to the control (stromal) value. (TIF) [file pone.0171004.s002.tif]

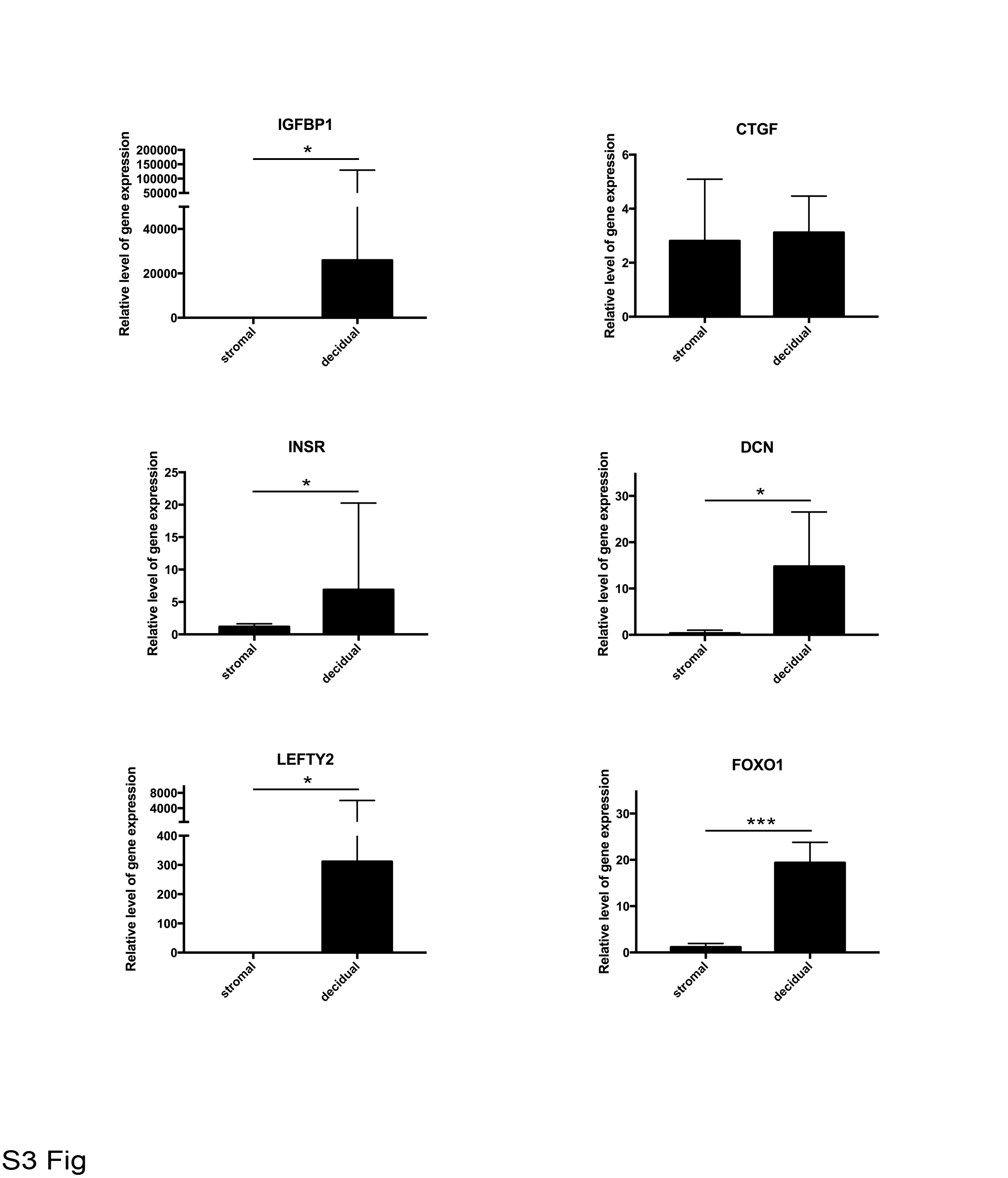

Supplement: S3 Fig — Relative gene expression levels of IGFBP1, CTGF, INSR, DCN, LEFTY2 and FOXO1 in the absence or presence of decidualization agents MPA (1 μM) and db-cAMP (0.5 mM) in endometrial stromal/decidual cells after 6 days. The values presented are medians and ranges (min-max). * = p < 0.05 and *** = p < 0.001 in comparison to the control value. (TIF) [file pone.0171004.s003.tif]

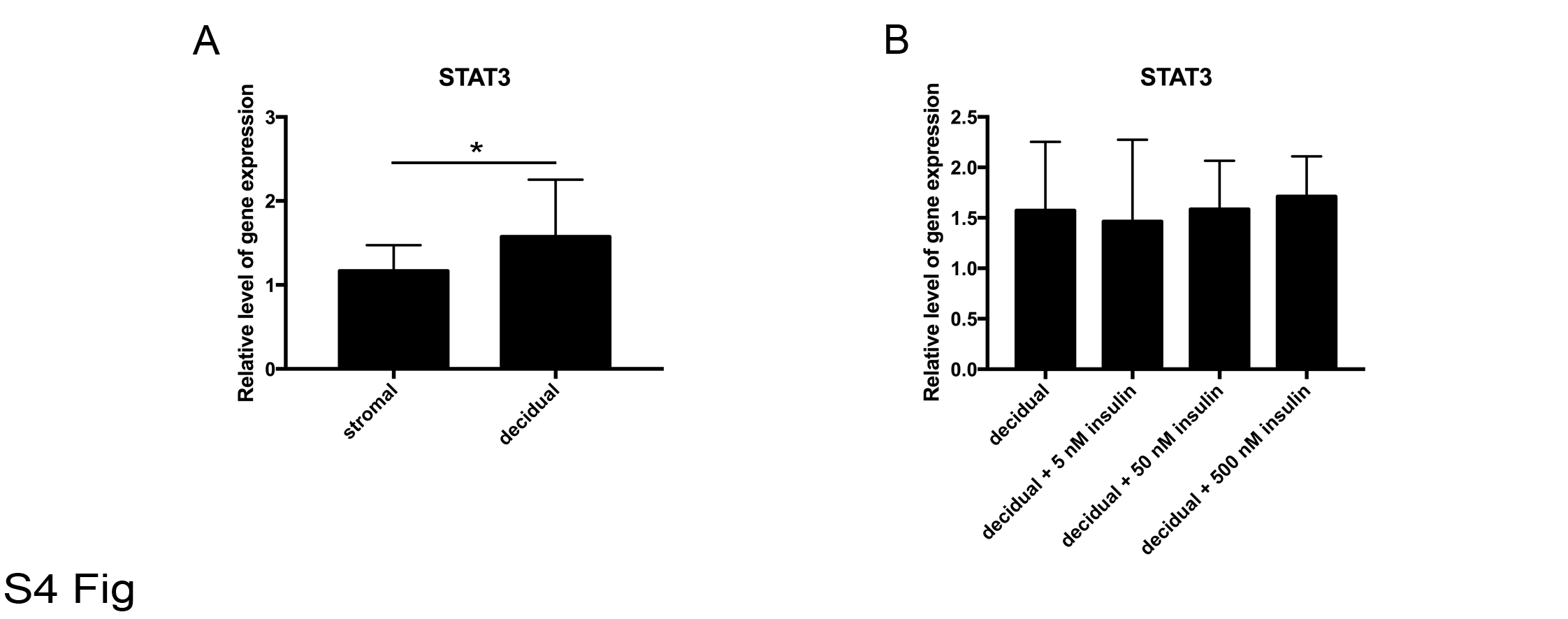

Supplement: S4 Fig — A. Relative gene expression level of STAT3 in the absence or presence of decidualization agents MPA (1 μM) and db-cAMP (0.5 mM) in endometrial stromal/decidual cells after 6 days. The values presented are medians and ranges (min-max). * = p < 0.05 in comparison to the control value. B. Relative gene expression level of STAT3 in response to decidualization agents MPA (1 μM) and db-cAMP (0.5 mM) in the presence or absence of 5, 50 and 500 nM insulin in endometrial stromal cells after 6 days. The values presented are medians and ranges (min-max). (TIF) [file pone.0171004.s004.tif]
