# Supplementary material for: Dysregulation of In Vitro Decidualization of Human Endometrial Stromal Cells by Insulin via Transcriptional Inhibition of Forkhead Box Protein O1
Source: PLoS One. 2017 Jan 30;12(1):e0171004. doi: 10.1371/journal.pone.0171004 (PMC5279782; doi:10.1371/journal.pone.0171004)
Supplement: S1 Table — TaqMan assays applied for amplification of prolactin (PRL), insulin-like growth factor binding protein-1 (IGFBP1) and insulin receptor (INSR). RPL13A was used as an endogenous control. (DOCX) [file pone.0171004.s005.docx]

**S1 Table.** TaqMan assays applied for amplification of prolactin (*PRL*), insulin-like growth factor binding protein-1 (*IGFBP1*) and insulin receptor (*INSR*). *RPL13A* was used as an endogenous control.

| Gene | TaqMan assay | NCBI reference sequence |
| --- | --- | --- |
| *PRL* | Hs00168730_m1 | NM_000948.5; NM_001163558.2 |
| *IGFBP1* | Hs00236877_m1 | NM_000596.2 |
| *INSR* | Hs00961554_m1 | NM_000208.2; NM_001079817.1 |
| *RPL13A* | Hs01926559_g1 | NM_012423.3; NM_001270491.1; NR_073024.1 |
