# Supplementary material for: Dysregulation of In Vitro Decidualization of Human Endometrial Stromal Cells by Insulin via Transcriptional Inhibition of Forkhead Box Protein O1
Source: PLoS One. 2017 Jan 30;12(1):e0171004. doi: 10.1371/journal.pone.0171004 (PMC5279782; doi:10.1371/journal.pone.0171004)
Supplement: S2 Table — Forward and reverse oligos applied for amplification of connective tissue growth factor (CTGF), decorin (DCN) and left-right determination factor 2 (LEFTY2). RPL13A was used as an endogenous control. (DOCX) [file pone.0171004.s006.docx]

**S2 Table.** Forward and reverse oligos applied for amplification of connective tissue growth factor (*CTGF*), decorin (*DCN*), left-right determination factor 2 (*LEFTY2*), forkhead box protein O1 (*FOXO1*) and signal transducer and activator of transcription 3 (*STAT3*). *RPL13A* was used as an endogenous control.

| Gene | Sequence | NCBI Reference Sequence |
| --- | --- | --- |
| *CTGF* | 5` -GCA GGC TAG AGA AGC AGA GC- 3` | NM_001901.2 |
|  | 5` -TGG AGA TTT TGG GAG TAC GG- 3` |  |
| *DCN* | 5` -TGG CAA CAA AAT CAG CAG AG- 3` | NM_133503.3; NM_133504.2; |
|  | 5` -GCC ATT GTC AAC AGC AGA GA- 3` | NM_133505.2 |
| *LEFTY2* | 5` -CCC TGG ACC TCA GGG ACT AT- 3` | NM_001172425.1; NM_003240.3 |
|  | 5` -CAG TTC TTG GCC CCA CTT CAT- 3` |  |
| *FOXO1* | 5` -AAG AGC GTG CCC TAC TTC AA - 3` | NM_002015.3 |
|  | 5` - TTC CTT CAT TCT GCA CAC GA- 3` |  |
| *STAT3* | 5`-GAG CTG GCT GAC TGG AAG AG- 3 | NM_003150.3 |
|  | 5`-TGT TGA CGG GTC TGA AGT TG- 3 |  |
| *RPL13A* | 5` -CAG GTC CTG GTG CTT GAT G - 3` | NM_012423.3; NR_073024.1 |
|  | 5` - GTT GAT GCC TTC ACA GCG TA- 3` |  |
